# Intermediate Charcot-Marie-Tooth disease due to a novel Trp101Stop myelin protein zero mutation associated with debilitating neuropathic pain

**DOI:** 10.1016/j.pain.2012.05.015

**Published:** 2012-08

**Authors:** Juan D. Ramirez, Phillip R.J. Barnes, Kerry R. Mills, David L.H. Bennett

**Affiliations:** a Wolfson Centre for Age-Related Diseases, King’s College London, London, UK; b Medical Director’s Office, Western Sussex Hospitals, Worthing, UK; c Department of Clinical Neurosciences, King’s College, London, UK

**Keywords:** Peripheral neuropathy, MPZ, Charcot-Marie-Tooth disease, Neuropathic pain, Quantitative sensory testing, Intraepidermal nerve fibre density

## Abstract

We report an English kindred affected across 4 generations with a hereditary neuropathy associated with debilitating neuropathic pain as the main clinical feature. The principal finding on clinical examination was sensory loss, and there was variable motor dysfunction. Electrophysiological studies revealed mild features of demyelination with median conduction velocity in the intermediate range. There was an autosomal-dominant pattern of inheritance, and genetic testing revealed a novel heterozygous Trp101X mutation in exon 3 coding for a portion of the extracellular domain of myelin protein zero. This is predicted to lead to premature termination of translation. Myelin protein zero is a key structural component of compact myelin, and over 100 mutations in this protein have been reported, which can give rise to neuropathies with either axonal, demyelinating, or intermediate features encompassing a wide range of severity. Chronic pain is an increasingly recognised sequela of certain hereditary neuropathies and may be musculoskeletal or neuropathic in origin. In this kindred, the neuropathy was relatively mild in severity, however, neuropathic pain was an important and disabling outcome.

## TOC summary

Inherited neuropathies present with striking variety in their phenotype. We present a family with a myelin protein zero mutation and disabling neuropathic pain.

## 1 Introduction

Certain hereditary neuropathies have a strong association with neuropathic pain. Classically, such neuropathies demonstrate sensory involvement and, particularly, injury to dorsal root ganglion neurons with unmyelinated axons. Examples include Fabry disease [36], familial amyloid neuropathy [22], and hereditary neuropathies due to mutations in serine palmitoyltransferase, long-chain base subunit-1 (SPTLC-1) [4,11], or RAB7 [12,33]. Some forms of Charcot-Marie-Tooth disease (CMT) are due to mutations in myelin proteins and can manifest prominent sensory involvement, for instance, CMT4F, which is caused by mutations in the periaxin gene [10,31]. The mechanisms underlying neuropathic pain generation are less clear in the context of demyelinating neuropathies. Myelin protein zero (MPZ) is the major glycoprotein of peripheral nerve myelin, and homotypic interactions between the extracellular domains of MPZ are required for the compaction of myelin lamellae. A large number of different MPZ mutations have been reported in association with CMT and can include axonal, demyelinating, or intermediate forms of CMT, with differing levels of severity [29]. We describe a multigenerational English kindred in whom the presenting feature was neuropathic pain with mild or absent motor dysfunction. This was found to be due to an intermediate form of CMT as a consequence of a novel mutation in the MPZ gene.

## 2 Methods

### 2.1 Patients and clinical evaluation

All the patients were seen by D.L.H.B. and P.R.J.B. at the Neurology Department, King’s College Hospital; 2 patients consented to detailed quantitative sensory testing as part of the Pain in Neuropathy Study (National Research Ethics Service Ref. 10/H07056/35). Genetic testing was performed following informed consent, and sequencing of the MPZ gene was undertaken at the National Genetics Reference Laboratory, Manchester, UK. Nucleotide numbering is given according to the MPZ cDNA sequence with Genebank accession number D10537. Nerve conduction studies were performed using standard techniques. The Charcot-Marie-Tooth Disease Neuropathy Score (CMTNS), a validated scale for assessing impairment in CMT, was calculated as per Shy et al. [28]. This is a composite measurement that involves sensory and motor symptoms and examination findings, as well as electrophysiological parameters. A score of 10 or less indicates a mild neuropathy; between 11 and 20, the neuropathy is considered moderate; and over 21 is severe; 36 points is the maximum score. For calculation of the Medical Research Council motor sum score, power was graded as per the Medical Research Council power scale (0-5) in the following muscle groups: arm abduction at the shoulder, elbow flexion, wrist extension, abduction of the index finger, hip flexion, knee extension, and ankle dorsiflexion, so that the maximum score is 70.

### 2.2 Quantitative sensory testing

Quantitative sensory testing following informed consent was performed in the S1 dermatome territory bilaterally according to the German DFNS protocol [24]. This protocol includes 13 parameters measuring temperature detection and pain thresholds, mechanical detection and pain thresholds, mechanical pain sensitivity, signs of allodynia, and wind-up ratio among others, allowing characterisation of sensory profiles. The findings in the 2 evaluated patients were compared with a control Caucasian population from Germany by means of *z* scores in which the *z* score represents the result of a raw score minus the mean of the population, and this is further divided by the SD of the population [24]. *Z*-scores above or below ±1.96 SDs would represent hyper- or hyposensitivity and hyper- or hypoalgesia, depending on the evaluated parameter.

### 2.3 Skin biopsy for intraepidermal nerve fibre density (IENFD) analysis

Following informed consent, a 3-mm punch skin biopsy was taken from the lower leg, 10 cm above the lateral malleolus, in patients and normal controls after anaesthetic injection of 1% lidocaine. Skin samples were fixed using 2% periodate-lysine-paraformaldehyde and then preserved in sucrose before being blocked and further processed into 50-μm sections. Samples were randomly selected for staining using goat antitype IV collagen antibody (1:400; Millipore, Temecula, CA, USA) followed by Alexa 488 donkey antigoat (1:1000; Alexa Fluor, Invitrogen, Life Technologies, Grand Island, NY, USA) to mark the basal membrane, nerve fibres were stained using rabbit anti-PGP (protein gene product) 9.5 Ab (1:2000; Ultraclone Ltd, Yarmouth, Isle of Wight, UK) and Cy3 antirabbit (1:500; Jackson Immunoresearch, West Grove, PA, USA). Further analysis of myelinated structures in the skin was performed according to published literature [23] using rat antimyelin basic protein Ab (1:200; AbCam, Cambridge, MA, USA) and Alexa 488 anti-rat (1:1000; Alexa Fluor, Invitrogen). Images were taken using a Zeiss LSM 710 upright confocal microscope, with a Plan-Apochromat objective at 20× magnification (Carl Zeiss MicroImaging GmbH, Jena, Germany). Eighteen 2-μm-interval images were acquired each time and the maximum intensity projection tool was used. Control samples and the patient’s sample were analyzed by the same investigator (J.D.R.). Analysis was performed as per published guidelines [13]. PGP 9.5-positive nerve fibres crossing the basal membrane were counted and a measurement of the length of the sample was then obtained. IENFD counts are given in number of fibres per millimetre length of epidermis.

## 3 Results

### 3.1 Clinical features

**Fig. 1:**
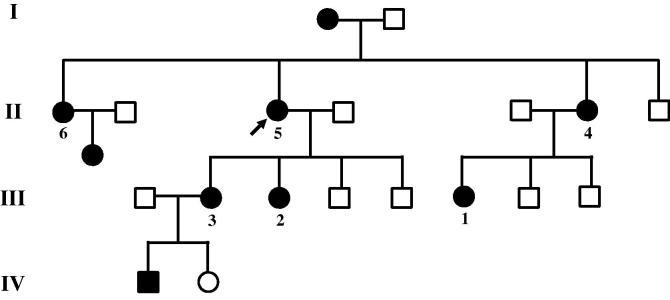
Family pedigree. Roman numerals represent the 1st to the 4th generation. Square, man; Circle, woman; Shaded symbol represents affected patients; Arrow points to the index case. Numbered subjects were seen by their health care team and are described in this report. Patients 5 and 2 were consented and entered the Pain in Neuropathy Study in which quantitative sensory testing was performed.

Six patients belonging to an English kindred over 4 generations (Fig. 1) were reviewed at the Neuromuscular Clinic, King’s College Hospital. In all cases, the presenting complaint was with sensory symptoms. All of the patients described pain that was localised to the toes, heels, and ankles, with a mean intensity on the numerical rating score of 7 of 10. The pain was described as being localised within deep structures of the ankle and foot. Descriptors included burning, aching, shooting, and throbbing pain. Pain was exacerbated by cold in 3 of the family members. Five of the 6 patients required medication for the management of their pain (including tricyclic antidepressants, gabapentinoids, opiates, and topical treatment with 5% lidocaine plaster), which were partially effective. Other sensory manifestations included numbness of the feet and intermittent paraesthesia. Only one patient complained of symptoms in the upper limbs, which consisted of intermittent paraesthesia. Motor symptoms were rare, however, one patient complained of weakness around the ankles.

On clinical examination (Table 1), findings in the cranial nerves were normal, including pupillary reactions. One patient had pes cavus. Power was normal in all the subjects except for a mild reduction in strength of ankle dorsiflexion in one subject. Sensory examination demonstrated in the majority of cases a distal, symmetrical sensory loss. None of the patients had touch-evoked allodynia. In most cases, ankle jerks were absent, but otherwise, deep tendon reflexes were preserved.

#### 3.1.1 Neurophysiology

Nerve conduction studies were performed in 4 of the affected subjects. The findings are summarised in Table 2 and demonstrated mild conduction slowing; the median nerve motor conduction velocity varied between 38 and 43 m/s, and f-waves were prolonged. The amplitudes of compound motor and sensory nerve action potentials were well maintained.

#### 3.1.2 Genetic analysis

In relation to genetic testing of the proband (Patient 5), molecular analysis of the PMP22 gene did not show duplication or deletion, and sequencing of the GJB1 gene did not reveal any mutations. Sequencing of the MPZ gene revealed a heterozygous G-to-A substitution at nucleotide 302 within exon 3. This is predicted to result in a premature termination of translation at codon 101. This codon normally encodes tryptophan, which is located in the extracellular domain of the protein [27]; the mutation would be predicted to result in a truncated protein lacking the transmembrane domain. All affected family members who were assessed were found to have this mutation.

### 3.2 CMTNS, quantitative sensory testing, IENFD measurements

The CMTNS was calculated in 4 patients and the score ranged between 6 and 9 (Table 1), which is indicative of a mild neuropathy [28].

**Fig. 2:**
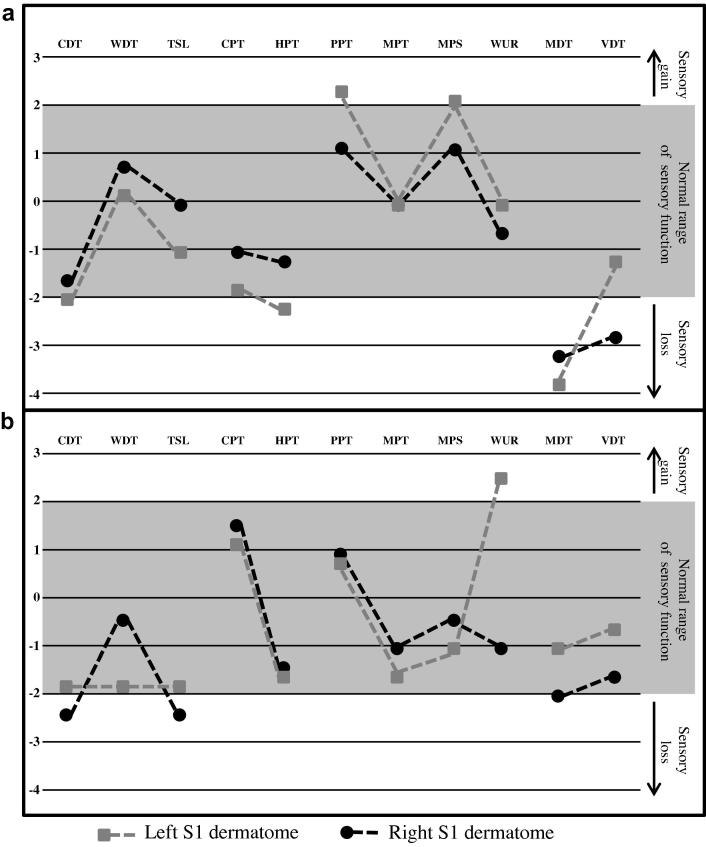
Quantitative sensory testing comparison of the lower limbs using *z* scores. (a) Patient 5. (b) Patient 2. Paradoxical heat sensations on Patient 5 were seen bilaterally (3 on each side); pain ratings did not show evidence of dynamic mechanical allodynia. CDT, cold-detection threshold; WDT, warm-detection threshold; TSL, thermal sensory limen; CPT, cold-pain threshold; HPT, heat-pain threshold; PPT, pressure-pain threshold; MPT, mechanical-pain threshold; MPS, mechanical-pain sensitivity; WUR, wind-up ratio; MDT, mechanical-detection threshold; VDT, vibration-detection threshold.

Quantitative sensory testing following the DFNS protocol was performed in 2 of the subjects. Findings can be seen in Fig. 2. *Z*-scores are compared against gender- and age-matched controls. Testing was performed bilaterally in the S1 dermatome, which was symptomatic in both patients, and generally, findings were symmetrical. There was evidence of sensory hypofunction, particularly in relation to cool-, mechanical-, and vibration-detection threshold. There was also some evidence for gain of function, for instance, lowered pressure pain threshold and increased wind-up ratio in individual patients.

**Fig. 3:**
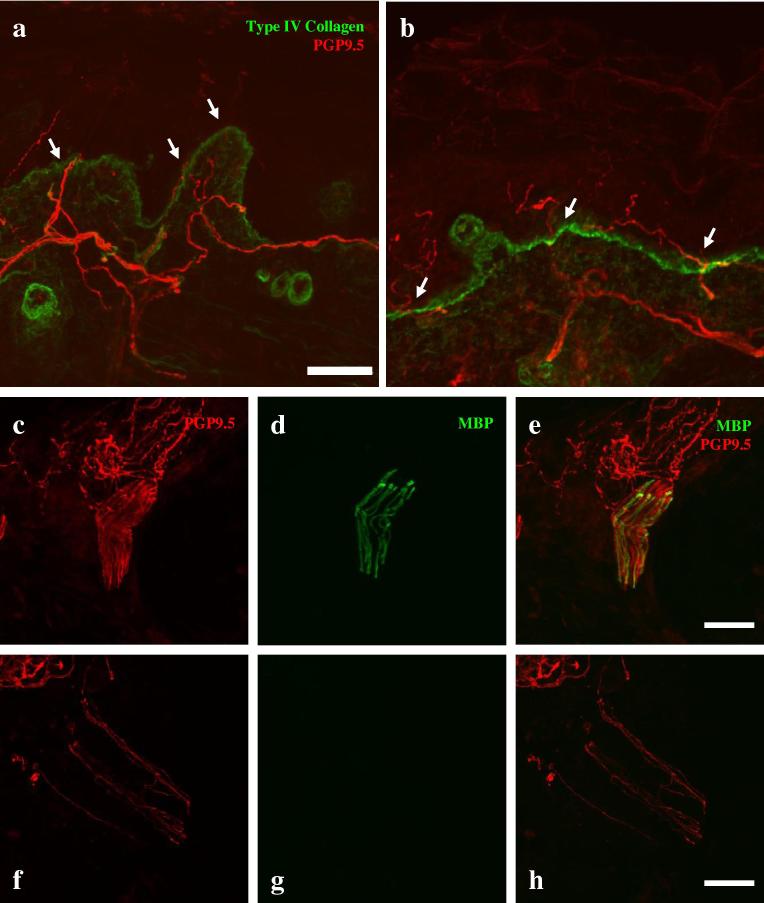
Immunohistochemistry. A maximum intensity projection of a confocal image of a lower-limb skin biopsy from a control subject (a) and Patient 5 (b) immunostained for protein gene product (PGP) 9.5 (red) to demonstrate axons and collagen type 4 (green) to show the basement membrane of the epidermis. There is no difference in the number of intraepidermal nerve fibres (arrows). (c-h) Confocal images of the dermis immunostained for PGP 9.5 [(c, f) red] and myelin basic protein [(d, g) MBP, green]. In Patient 5 (f, g, h), although numerous axons can be seen, there are no myelinated internodes, while in a control subject (c, d, e), numerous myelinated internodes were observed. Scale Bar: 50 μm.

Skin biopsy of the lower leg was performed in Patient 5. There was no evidence of loss of intraepidermal nerve fibres. The intraepidermal nerve fibre density was normal, at 10.67 fibres/mm (relative to a mean of 10.13 ± 2.6 fibres/mm in control for this laboratory) (Fig. 3). In control subjects, as has previously been demonstrated by Provitera et al. [23], immunostaining for both PGP 9.5 and myelin basic protein revealed bundles of axons with compact myelin within the dermis (Fig. 3). Despite examination of multiple sections derived from the skin biopsy of Patient 5, although many axons could be visualised within the dermis, none of them were myelinated.

## 4 Discussion

To summarise, we report a kindred with a novel mutation in the MPZ gene, which results in a mild form of CMT disease (in relation to motor dysfunction and sensory loss), but debilitating neuropathic pain.

MPZ is an integral membrane glycoprotein synthesised by Schwann cells; it is the major glycoprotein of peripheral nerve myelin and possesses an intracellular and extracellular domain. Homotypic interactions between the extracellular domains of MPZ mediate the compaction of myelin lamellae [6,16,18]. A large number of different MPZ mutations have been reported in association with CMT (Inherited Peripheral Neuropathies Mutation Database: Mutations in MPZ [P0] http://www.molgen.ua.ac.be/CMTMutations/Mutations/Mutations.cfm?Context=2). These display striking phenotypic variability and include axonal, demyelinating, and intermediate forms of CMT, with differing levels of severity from mild to severe neuropathies [29]. These can be broadly split into 2 groups: a severe early-onset demyelinating neuropathy and a late-onset neuropathy with predominantly axonal loss. Certain mutations associated with the former have gain of function-dominant negative effects and mutations associated with the latter partial loss of function [9]. The Trp101X mutation is predicted to result in premature termination of translation and a truncated protein lacking the transmembrane domain required for membrane anchorage. This may, therefore, be predicted to lead to less MPZ protein insertion into the myelin membrane. The level of MPZ within myelin membranes was found to be normal in another mutation that causes premature truncation: the heterozygous Val102 frame shift mutation [30]. This indicates that there may be potential compensatory mechanisms, although such compensation for gene dosage must be incomplete, as individuals with these mutations develop a mild neuropathy.

CMT is increasingly recognised as a cause of both nociceptive and neuropathic pain [3,7], and there is some evidence that neuropathic pain may be more common in the axonal rather than demyelinating forms of CMT. The distribution and nature of the pain experienced by patients with the Trp101X MPZ mutation was neuropathic in nature, and these patients would fulfil recent criteria for definite neuropathic pain [32]. There have been occasional reports of neuropathic pain associated with MPZ mutations. Burns et al. reported a patient who developed an acute neuropathy with pain as a prominent feature, evidence of demyelination on neurophysiology, who was found to have an Arg36Trp mutation in MPZ [2]. Schneider-Golf et al. report a German kindred with an Asp234Tyr missense mutation resulting in a neuropathy with features of intermediate conduction, the predominant symptom being burning sensations in the arms and legs as well as muscle cramps [26]. Such reports are, however, the exception rather than the rule.

A number of other frame shift or premature stop MPZ mutations leading to a nonfunctional truncated MPZ protein in the heterozygous state (ie, those mutations likely to share the same pathophysiological mechanisms as the Trp101x mutation) have been described as causing a mild neuropathy with intermediate conduction. The symptoms are often predominantly sensory rather than motor, including paraesthesia and muscle cramps; and the index case carrying the Glu71stop mutation described by Lagueny et al. complained of burning pain of the extremities [15,20,21,35]. It should be noted that some subjects carrying such mutations have, however, been reported to be asymptomatic, indicating potential modifying factors.

Extensive preclinical literature exists regarding the aetiology of neuropathic pain following traumatic nerve injury or in the context of axonal neuropathies [19]. Less is known regarding the pathophysiology of neuropathic pain in demyelinating neuropathies. Primary demyelination has been shown to induce neuropathic pain-related hypersensitivity that was associated with ectopic activity in primary afferents [34]. Mice that lack the myelin constituent periaxin have been shown to develop ectopic activity in primary afferents and pain-related hypersensitivity [8] associated with demyelination. The generation of neuropathic pain by demyelination may relate to a number of potential mechanisms, including the redistribution of ion channels in demyelinated axolemma [25], Schwann cell proliferation [1], the release of cytokines and chemokines [14], and the recruitment of inflammatory cells such as macrophages [17]. The neuropathic pain reported in relation to the MPZ mutations described by Burns et al. and Schneider-Gold et al. was thought to partially have an immune aetiology, given the acute onset, and in one case, the patient’s pain responded to treatment with intravenous immunoglobulin [2,26].

The majority of our patients with the Trp101X mutation had evidence of distal, symmetrical sensory impairment on bedside testing. Only 2 patients agreed to detailed quantitative sensory testing, making it difficult to draw definitive conclusions about the pattern of sensory dysfunction in these patients. Both patients had a tendency to raised detection thresholds to cooling, which is likely to relate to impaired function in thin myelinated fibres [5]. In relation to the function of large myelinated sensory fibres, one patient had raised mechanical-detection threshold in response to the application of von Frey filaments and impaired vibration detection. There were also some signs of hypersensitivity to sensory stimuli applied to the foot, including a unilateral enhanced wind-up ratio and lowered pressure pain threshold. In one patient who consented to a skin biopsy, there was no evidence of loss of C fibres within the epidermis of the lower leg (a region that was symptomatic in relation to pain). We had hoped to assess whether there was evidence of demyelination and altered ion channel distribution in myelinated dermal fibres. Myelinated internodes have previously been reported in skin biopsy samples [23] and were reliably observed in our control samples. We could not, however, visualise any myelinated internodes in the patient with the Trp101X MPZ mutation despite immunostaining of sections throughout the sample. This may be indicative of distal demyelination; however, we cannot exclude the possibility that this could be a sampling issue. It is likely that dysfunction in sensory axons that would normally be myelinated is responsible for the neuropathic pain in these patients.

These cases emphasise the heterogeneity of the phenotype of MPZ mutations, the fact that mutations in myelin proteins can result in disabling neuropathic pain and that there is no simple relationship between overall neuropathy severity and the degree of neuropathic pain.

## Conflict of interest statement

No conflict of interest is reported.

## Figures and Tables

**Table 1:** Clinical features of the reported kindred.

	Age of onset	Age at assessment, y	Pain	Pes cavus	Examination					CMTNS
					MRC sum score (max 70)	Pin prick	Light touch	Joint position sense	Vibration sense	
Patient 1	1st decade	18	Yes	No	70	Normal	Normal	Normal	Normal	∗
Patient 2	1st decade	23	Yes	No	68	Normal	Normal	Normal	Normal	7
Patient 3	2nd decade	25	Yes	No	70	Lost to the ankles	Normal	Normal	Lost to the toes	8
Patient 4	3rd decade	40	Yes	No	70	Lost to wrists and knees	Normal	Normal	Lost to the toes	9
Patient 5	3rd decade	42	Yes	No	70	Lost to the ankles	Normal	Normal	Normal	6
Patient 6	4th decade	44	Yes	Yes	70	Lost to the ankles	Lost to the toes	Lost to the toes	Lost to the toes	∗

MRC sum score, summation of the Medical Research Council (MRC) grades from 0 to 5 in bilaterally tested muscles (max. score of 70); CMTNS, Charcot-Marie-Tooth disease Neuropathy Score, which has a maximum score of 36. Age of pain onset ranged from the 1st to the 4th decade. All the patients reported pain as their presenting complaint.

**Table 2:** Nerve conduction studies done as part of the clinical assessment.

	Sensory studies					Motor studies				
		R Median	R Ulnar	R Radial	R Sural		R Median	L Median	R Ulnar	Peroneal
Patient 2	SNAP (μV)	14.6	5.8	17.2	16.4	CMAP (mV)	8.7	NT	7.7	3.1
	CV (m/s)	48	43	50	42	DML (ms)	4.5	NT	2.8	5.1
						CV (m/s)	43	NT	44	39
						F-Latency (m/s)	32.1	NT	32.9	57.1

Patient 3	SNAP (μV)	12	10.1	NT	19.4	CMAP (mV)	10.2	9.5	5.4	NT
	CV (m/s)	38	40	NT	33	DML (ms)	3.6	3.7	3	NT
						CV (m/s)	39	39	38	NT
						F-Latency (m/s)	31	30.9	32.3	NT

Patient 5	SNAP (μV)	10.4	10.3	NT	NT	CMAP (mV)	5.6	1.8	2.2	NT
	CV (m/s)	43	42	NT	NT	DML (ms)	4.9	5.4	4.2	NT
						CV (m/s)	41	33	50	NT
						F-Latency (m/s)	NT	NT	NT	NT

Patient 6	SNAP (μV)	16.9	18	48.7	13.4	CMAP (mV)	8.5	NT	NT	3.6
	CV (m/s)	37	33	42	35	DML (ms)	4.8	NT	NT	4.3
						CV (m/s)	38	NT	NT	36
						F-Latency (m/s)	35.6	NT	34.9	NT

R, right; L, left; SNAP, sensory nerve action potential; CV, conduction velocity; CMAP, compound motor action potential; DML, distal motor latency; F-Latency, F-wave latency; NT, not tested.
